# Differences in APOBEC3G Expression in CD4+ T Helper Lymphocyte Subtypes Modulate HIV-1 Infectivity

**DOI:** 10.1371/journal.ppat.1000292

**Published:** 2009-02-06

**Authors:** Michael L. Vetter, Megan E. Johnson, Amanda K. Antons, Derya Unutmaz, Richard T. D'Aquila

**Affiliations:** 1 Department of Microbiology and Immunology, Vanderbilt University, Nashville, Tennessee, United States of America; 2 Department of Microbiology, New York University, New York, New York, United States of America; 3 Department of Medicine, Division of Infectious Diseases, Vanderbilt University, Nashville, Tennessee, United States of America; NIH/NIAID, United States of America

## Abstract

The cytidine deaminases APOBEC3G and APOBEC3F exert anti–HIV-1 activity that is countered by the HIV-1 vif protein. Based on potential transcription factor binding sites in their putative promoters, we hypothesized that expression of APOBEC3G and APOBEC3F would vary with T helper lymphocyte differentiation. Naive CD4+ T lymphocytes were differentiated to T helper type 1 (Th1) and 2 (Th2) effector cells by expression of transcription factors Tbet and GATA3, respectively, as well as by cytokine polarization. APOBEC3G and APOBEC3F RNA levels, and APOBEC3G protein levels, were higher in Th1 than in Th2 cells. T cell receptor stimulation further increased APOBEC3G and APOBEC3F expression in Tbet- and control-transduced, but not in GATA3-transduced, cells. Neutralizing anti–interferon-γ antibodies reduced both basal and T cell receptor-stimulated APOBEC3G and APOBEC3F expression in Tbet- and control-transduced cells. HIV-1 produced from Th1 cells had more virion APOBEC3G, and decreased infectivity, compared to virions produced from Th2 cells. These differences between Th1- and Th2-produced virions were greater for viruses lacking functional *vif*, but also seen with *vif*-positive viruses. Over-expression of APOBEC3G in Th2 cells decreased the infectivity of virions produced from Th2 cells, and reduction of APOBEC3G in Th1 cells increased infectivity of virions produced from Th1 cells, consistent with a causal role for APOBEC3G in the infectivity difference. These results indicate that APOBEC3G and APOBEC3F levels vary physiologically during CD4+ T lymphocyte differentiation, that interferon-γ contributes to this modulation, and that this physiological regulation can cause changes in infectivity of progeny virions, even in the presence of HIV-1 vif.

## Author Summary

Some host cell proteins can hinder, or restrict, the life cycle of HIV-1. APOBEC3G and APOBEC3F are cellular enzymes that decrease HIV-1's ability to replicate in a subsequent target cell if they are present in the virus particle. As a countermeasure, HIV-1 virion infectivity factor (*vif*) induces degradation of APOBEC3G and APOBEC3F, thereby preventing them from getting into the budding virus. Although *vif*-defective viruses cannot evade the antiviral effect of APOBEC3G, such viruses are very rarely present in HIV-1-infected humans. It is not yet known whether physiological variation in APOBEC3G and APOBEC3F expression in CD4+ T lymphocytes is substantial enough to reduce *vif*-positive HIV-1 infectivity. In this study, we found that T helper type 1 (Th1) cells, a subtype of CD4+ lymphocytes, expressed greater amounts of APOBEC3G and APOBEC3F than T helper type 2 (Th2) cells. This difference led to a difference in infectivity of HIV-1 produced from the two cell types, whether *vif* was expressed or not. These results demonstrate that physiological regulation of APOBEC3G does restrict *vif*-positive HIV-1, as well as *vif*-negative HIV-1. In addition, this study reveals biological factors regulating expression of these proteins that may be exploitable for new therapeutic or preventive strategies against HIV-1.

## Introduction

APOBEC3G (hA3G) and APOBEC3F (hA3F), two of several related cytidine deaminases, evolved to limit retrotransposition [1]–[3]. Although the HIV-1 accessory protein vif depletes hA3G and hA3F from the producer cell, hA3G and hA3F are packaged into *vif*-deleted HIV-1 and significantly impair virion infectivity [4]–[6]. IFN-α, and certain cytokines and mitogens, have been implicated in increasing hA3G and hA3F expression in certain cell types [7]–[12]. However, little more is known regarding the transcriptional regulation of APOBEC3s in CD4+ T lymphocytes [13]. We noted several potential binding sites for GATA family transcription factors [14], in addition to previously observed interferon-responsive elements [7],[12],[15], in the putative promoter regions of hA3G and hA3F. Since GATA3 is integral to the differentiation of naïve CD4+ T helper cells into Type 2 (Th2) effectors, we hypothesized that Type 1 (Th1) and Th2 effector lymphocytes differed in their expression of hA3G and hA3F.

After naive CD4+ T lymphocytes interact with their cognate antigen, IL-12 and interferon-γ (IFN-γ) signaling drive their differentiation to a Th1 effector phenotype. In contrast, IL-4 signaling after antigen recognition drives differentiation of naïve cells to a Th2 phenotype [16]–[18]. These subtypes of T helper cells produce distinct cytokine profiles after subsequent activation. Th1 cells, when activated, produce IFN-γ to activate cell-mediated immunity. Th2 cells, however, secrete IL-4 and other cytokines which augment humoral immune responses. The differentiation to a Th1 or Th2 phenotype is dependent on the regulated expression of two master transcriptional regulators, respectively: T Box expressed in T cells (Tbet) and GATA3 [19]–[21]. Relative differences in the ability of Th1 versus Th2 subtypes to produce infectious wild-type HIV-1 progeny have been reported previously in several studies and were not explained by differences in expression of chemokine co-receptors for HIV entry [22]–[25].

Although high level over-expression of hA3G has been reported to decrease infectivity of *vif*-positive virions produced from cell lines *in vitro* [4], [26]–[28], it is not known whether physiological increases in hA3G or hA3F can overcome the effect of vif in primary T cells. Reports conflict about whether differences in levels of hA3G and hA3F in lymphocytes *in vivo* are inversely associated with the level of wild-type HIV-1 RNA in plasma of untreated patients [29]–[31]. One of two reports has correlated provirus hypermutation attributable to hA3G and hA3F with plasma viral load, consistent with effects *in vivo* against at least some *vif*-positive viruses [32],[33]. Since an effect of variation of levels of hA3G and hA3F in a physiologically relevant range on wild-type, *vif*-positive HIV-1 replication has not yet been directly demonstrated, the present study aimed to define if such cellular differences occur during Th1 versus Th2 differentiation and may cause changes in HIV-1 infectivity that affect pathogenesis.

## Results

### Master Transcriptional Regulators Modulate APOBEC3G and APOBEC3F Expression

**Figure 1:**
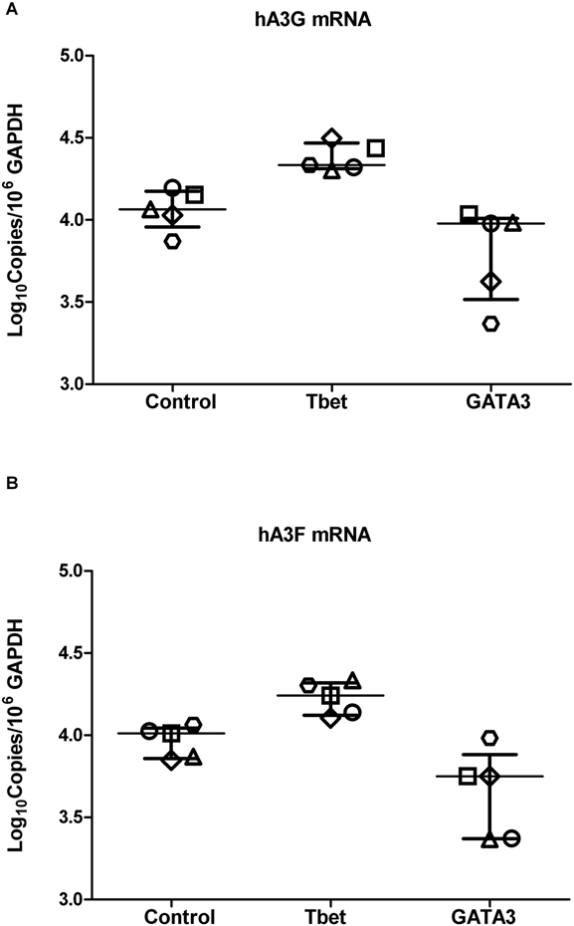
Tbet and GATA3 regulate hA3G and hA3F expression. Naïve CD4+ T cells were transduced with a Tbet or GATA3 expressing lentiviral vector. After sorting based on GFP marker gene expression, cytoplasmic RNA was isolated and used to determine mRNA levels of hA3G (A) and hA3F (B) by qRT-PCR. Data are expressed as copy number of hA3G or hA3F per 10^6^ copies of GAPDH. Error bars represent median and interquartile range.

Naïve CD4+ T cells from five individual HIV-1 negative donors were transduced with HIV-derived lentiviral vectors that expressed either GFP alone (control), or together with Tbet or GATA3. Expression of GATA3 and Tbet were found to have opposing effects on the expression of hA3G and hA3F mRNA by qRT-PCR (Figure 1A and 1B). Whereas expression of GATA3 reduced the level of hA3G and hA3F, Tbet significantly increased the levels of both enzymes. Based on these results, confirmation that this was a statistically and biologically significant effect was sought by studying Th1 versus Th2 differentiation using more physiological cytokine polarization.

### CD4+ T Helper Type 2 Lymphocytes Express Lower Levels of APOBEC3G and APOBEC3F than T Helper Type 1 Lymphocytes

**Figure 2:**
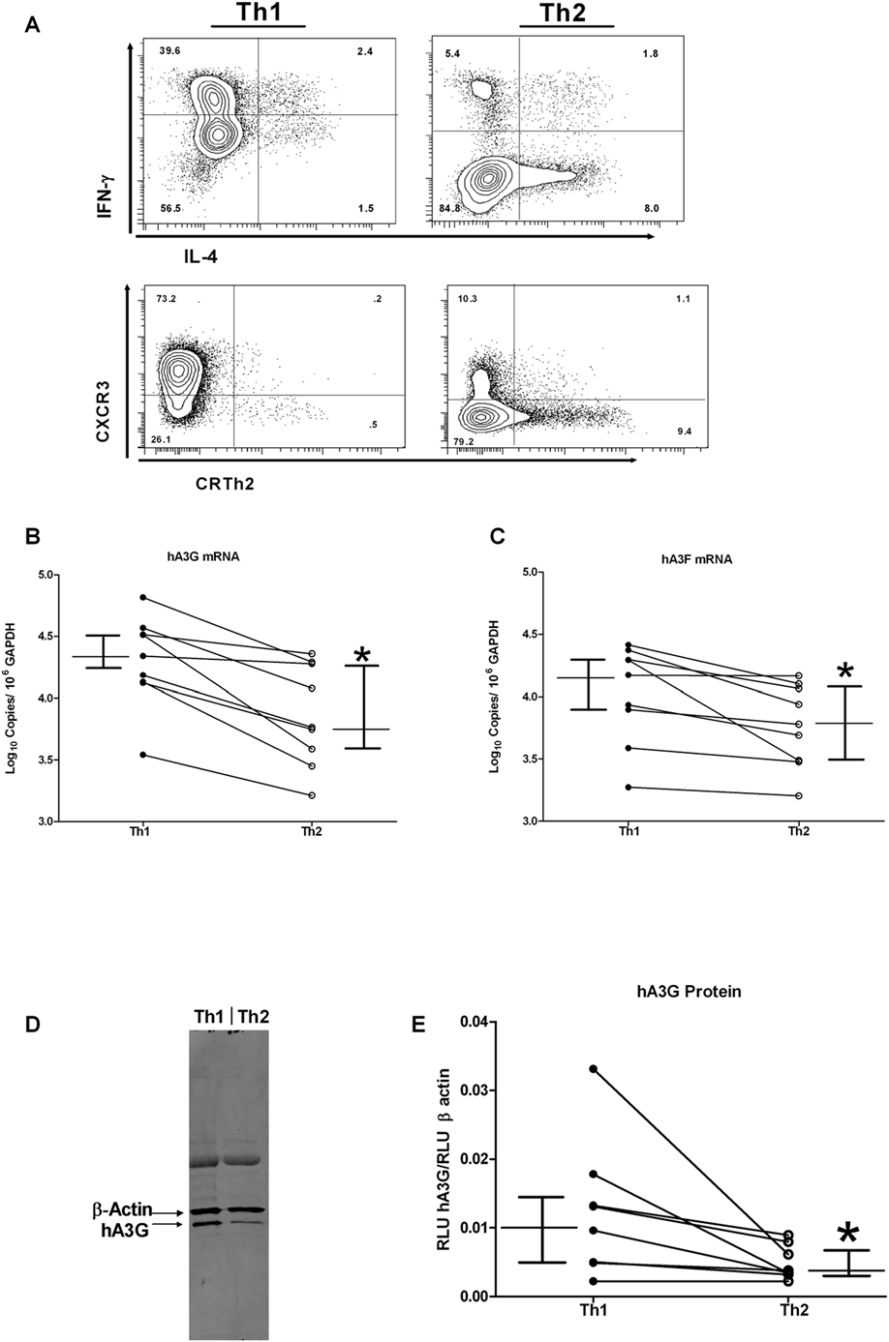
Th2 cells express lower levels of hA3G and hA3F than Th1. Naïve CD4+ T cells were derived to either a Th1 or Th2 phenotype using cytokines as described in Materials and Methods. The cells were then stained for intracellular cytokine production or surface markers (A) to confirm differentiation. Cytoplasmic RNA was isolated from the cells and used for qRT-PCR to determine the level of hA3G (B) or hA3F (C) mRNA. Error bars represent media and interquartile range (*p = 0.0039). *In vitro* cytokine-derived Th1 or Th2 cells were also lysed and subjected to Western Blotting with a hA3G specific antibody and levels of expression were quantified using a LICOR Odyssey system. A representative blot is shown (D) as well as the compilation of 8 individual donors (E) with quantities expressed as quantified intensity of hA3G bands per quantified intensity of Beta-Actin bands of the same lane. Error bars represent median and interquartile range (*p = 0.0078).

Th1 and Th2 cells were differentiated *in vitro* by culturing naïve cells from nine individual donors in polarizing cytokines. Staining for Th1- and Th2-associated intracellular cytokines (IFN-γ and IL-4, respectively) and surface markers (CXCR3 and CRTh2, respectively) (Figure 2A), verified the phenotypes of the cytokine-differentiated cells. Cytoplasmic RNA was isolated and the levels of hA3G and hA3F mRNA were determined relative to GAPDH expression by qRT-PCR. Th2 cells expressed significantly less hA3G and hA3F mRNA than Th1 cells (Figure 2B and 2C). Western blot analysis of the two helper cell subtypes revealed that Th2 cells also expressed lower levels of hA3G protein than Th1 cells (Figure 2D and 2E). The statistically significant difference in median hA3G mRNA levels, and in protein levels, between Th1 and Th2 cells was approximately 3-fold.

### Interferon-γ Regulates Basal and TCR-Stimulated Expression of APOBEC3s in Tbet-Transduced Cells

**Figure 3:**
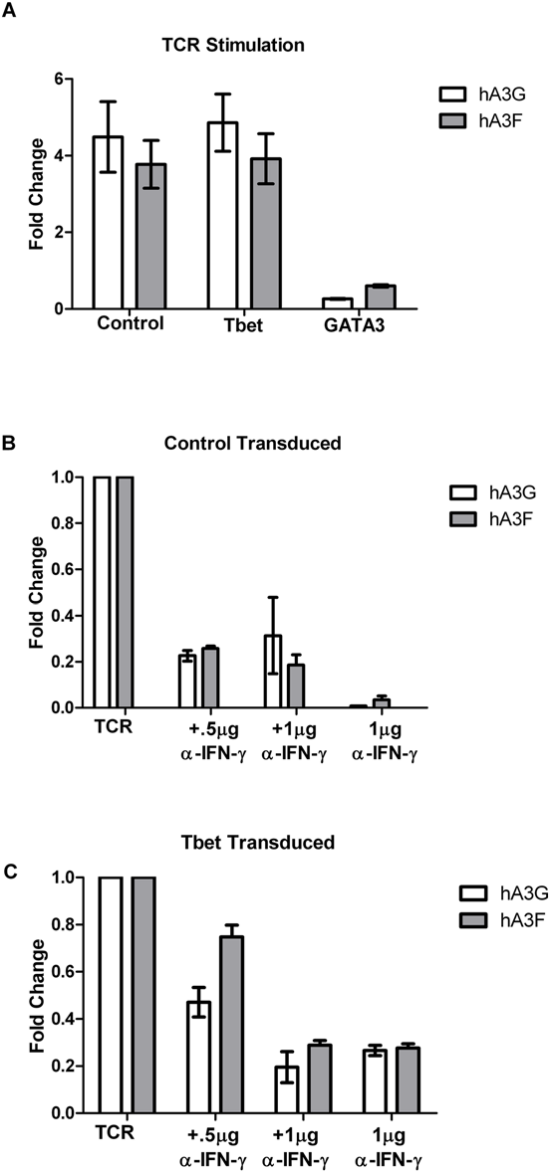
Interferon gamma regulates expression of hA3G and hA3F in Tbet but not GATA3 transduced cells. Control, Tbet, and GATA3 transduced cells were TCR stimulated with CD3/CD28 beads. Cytoplasmic RNA was then isolated to determine the fold change in mRNA expression by qRT-PCR (A). Control (B) and Tbet (C) transduced cells were TCR stimulated or left unstimulated in the presence of a neutralizing anti-interferon gamma antibody. The fold change in mRNA expression was again determined by qRT-PCR. Incubation of TCR stimulated cells with isotype control does not differ significantly from stimulation alone (data not shown). Error bars represent standard deviation from the mean.

Since previous studies have observed that mitogen treatment increases hA3G expression [7], we tested whether T Cell Receptor (TCR) stimulation would increase hA3G and hA3F expression in Tbet and GATA3 expressing T-cells. Levels of hA3G and hA3F RNAs increased after TCR stimulation of control vector- and Tbet-transduced cells, while this did not occur with TCR activation of GATA3-transduced cells (Figure 3A). A defining characteristic of Th1 cells is their ability to produce IFN-γ upon activation, which then exerts autocrine effects [34]. It is also known that GATA3 diminishes IFN-γ expression. Therefore, the hypothesis that IFN-γ contributes to the observed increase in hA3G and hA3F expression after TCR stimulation was tested by performing TCR stimulation of control- and Tbet-transduced cells in the absence or presence of a neutralizing anti- IFN-γ antibody. The presence of neutralizing anti- IFN-γ antibody blocked the TCR-stimulated increased transcription of hA3G and hA3F, and reduced basal levels, in both control- and Tbet-transduced cells (Figure 3B and 3C). This suggests that IFN-γ contributes to maintaining the steady state level of hA3G and hA3F in Th1 cells, as well as in increasing expression after TCR activation.

### Increased Infectivity of HIV-1 Produced from CD4+ T Helper Type 2 Compared to Type 1 Lymphocytes

**Figure 4:**
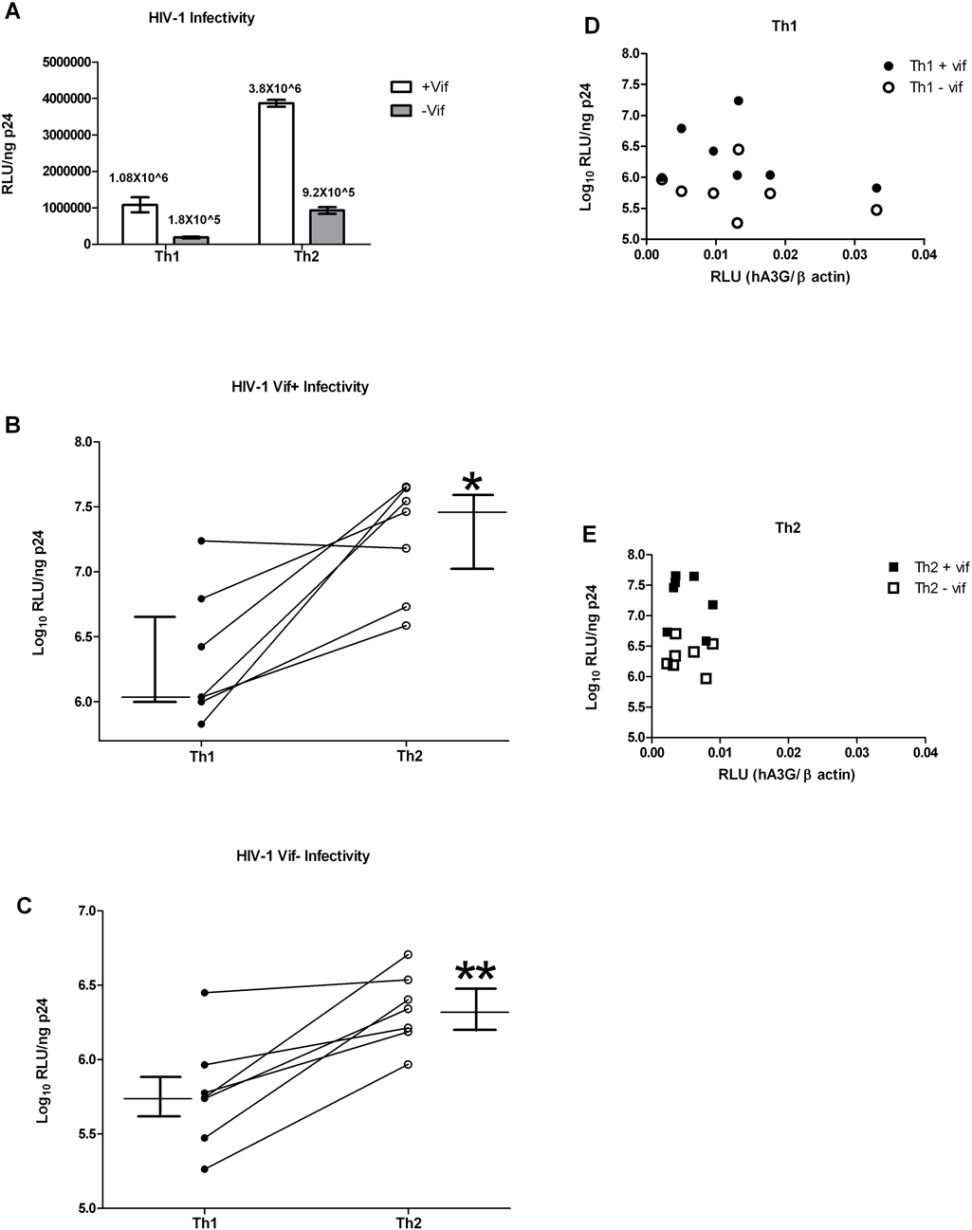
Increased Infectivity of HIV-1 produced from Th2 cells compared to Th1 cells. *vif*-competent (+) or *vif*-deleted (−) HIV-1(NL4-3) was used to infect cultures of Th1 and Th2 cells as described in Materials and Methods. Infectivity of the virions produced was determined by infection of the TZM-bl indicator cell line and determination of luciferase activity. An example from an individual donor is shown (A). The experiment was repeated on a total of seven donors with both the *vif*-competent (B) and *vif*-deleted virus(C). Error bars represent median and interquartile range of difference in infectivity of virions produced from Th1 cells versus Th2 cells for vif-positive virus (B; *p = .031) and vif-negative virus (C; **p = .016). To test for correlation, individual donor cells' levels of hA3G protein expression were plotted against infectivity of the *vif*-competent and *vif*-deleted virions produced from that individual's Th1 cells (D) and Th2 cells (E).

We next tested whether the differential expression of APOBEC3s between Th1 and Th2 cells led to a difference in infectivity of HIV-1 virions produced from these cells. We infected TCR-activated, cytokine-derived T helper cells with *vif*-deleted or *vif*-competent HIV-1(NL4-3) produced from 293T cells (which do not express hA3G or hA3F). Infected cells were washed 12 hours after infection and new media containing reverse transcriptase inhibitors (didanosine and zidovudine) was added to prevent spread past the first-round infected cells. Twelve hours after the new media was added, the culture supernatant fluids were collected, normalized by Gag p24 capsid antigen concentrations, and used to infect the TZM-bl indicator cell line. Infectivity was determined by luciferase activity. Figure 4A quantitates infectivity of wild-type and *vif*-deleted viruses produced from Th1 and Th2 cells from one of nine donors studied. *Vif*-negative viruses produced from Th2 cells from this individual were five-fold more infectious than those produced from Th1 cells, whereas *vif*-competent virions from Th2 cells were three-fold more infectious than those produced from Th1 cells (Figure 4A). The median infectivity of virions produced from Th1 cells of all nine donors studied was significantly less than that of viruses produced from all the different Th2 cells, whether vif was present or not (Figure 4B and 4C). The magnitude of this difference varied across different individual donors' paired Th1 and Th2 cells, whether vif was present or not (Figure 4B and 4C; each donor's Th1 and Th2 cells are linked by a line). A3G protein levels also varied, with Th1 cells having higher levels and a broader range of hA3G protein than Th2 cells (comparing X-axis in Figure 4D and 4E). Despite the small number of subjects and variability of the assays, there was a suggestion of an inverse correlation between A3G protein expression and infectivity of virions produced from Th1 (+vif r = −.16, −vif r = −.18; all p>0.05) or Th2 (+vif r = −.28, −vif r = −.01; all p>0.05). We amplified a pol gene fragment from the TZM-bl cells infected for 60 hours with Th1- or Th2-produced *vif*-negative virions to quantify if effects of cytidine deamination differed by cell type. Hypermutation was not seen in HIV pol DNA amplified from cells infected with virus produced from either cell type (data not shown), using either population sequencing subsequent to standard PCR or 3D PCR [35]. Although these data are consistent with direct effects of hA3G and hA3F on infectivity of *vif*-competent HIV as well as *vif*-defective HIV-1, it is possible that other variables may affect infectivity of virions produced from these cytokine-polarized cells.

### Changes in APOBEC3G Cause Differences in Infectivity of Th1- Versus Th2-produced HIV-1

**Figure 5:**
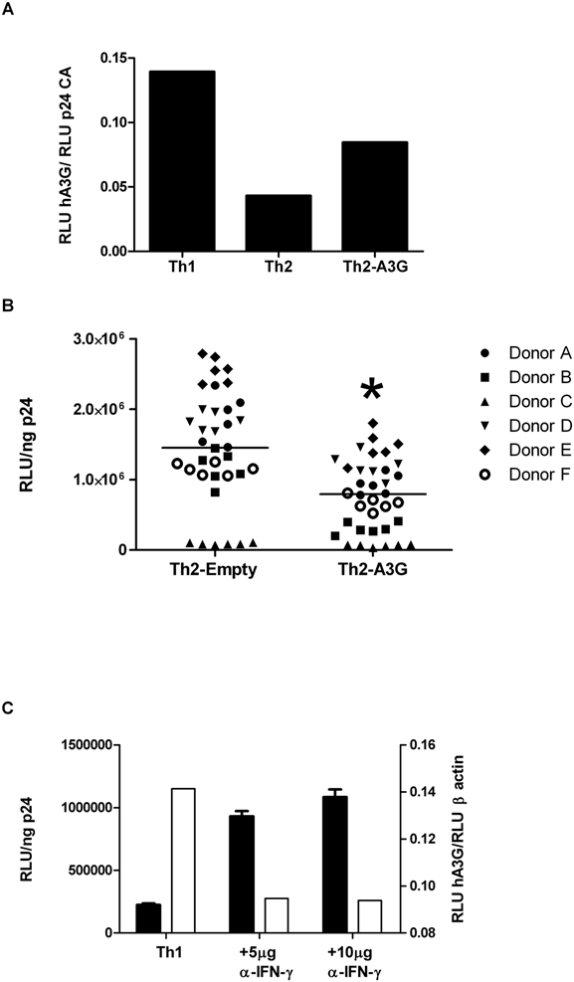
Evidence supporting a direct effect of hA3G on relative infectivity of HIV-1 virions produced from Th1 and Th2 cells. In vitro cytokine-derived Th2 cells were transduced with a control lentiviral vector (Th2-Empty) or a vector expressing hA3G (Th2-A3G). Protein levels of hA3G packaged into virions produced from Th1, Th2, and hA3G-transduced Th2 cells were determined by Western Blotting on the LICOR Odyssey system (Relative Light Units (RLU) of hA3G as normalized by HIV-1 p24 antigen) Shown is a representative blot of three experiments with similar trends. (A). The infectivity of *vif*-deleted HIV-1(NL4-3) virions produced from those cells from 6 donors was determined using the TZM-bl indicator cell line. Error bars represent median and interquartile range (*p = .03). (B) In vitro cytokine-derived Th1 cells were activated and incubated with either an isotype control or neutralizing IFN-γ antibodies for 48 hours. Protein concentrations were determined by Western blot (C,open bars). The infectivity of *vif*-deleted HIV-1(NL4-3) virions produced from those cells was determined using the TZM-bl indicator cell line (C, closed bars).

To confirm a causal role for hA3G in the observed virion infectivity differences we modulated expression of A3G in Th1 and Th2 cells by increasing expression in Th2 cells and decreasing expression in Th1 cells. We increased expression of hA3G in cytokine-derived Th2 cells by transduction with a hA3G-expressing lentiviral vector or an “empty” control vector for comparison. Transduction of Th2 cells with the hA3G-expressing vector increased hA3G levels 4 fold over Th1 cells and 7 fold over Th2 cells (data not shown). After expansion, the unsorted population of hA3G vector-transduced Th2 cells (Th2-A3G), as well as Th1 and Th2 cells, were infected. The *vif*-deleted virions produced from Th1, Th2 and Th2-A3G cells were concentrated and the relative levels of virion packaged hA3G were determined by Western blotting. Figure 5A demonstrates that *vif*-deleted virions produced from Th1 cells contain more hA3G than virions produced from Th2 cells. Th2-A3G cells produced virions with more packaged hA3G than Th2 cells (Figure 5A). Transduction with the empty vector (Th2-Empty) caused no increase in cellular or virion hA3G levels, relative to untransduced Th2 cells (data not shown). Virions produced from the Th2-A3G cells were significantly less infectious than those produced from the Th2 cells transduced with the “empty” control vector (“Th2-Empty”) (Figure 5B). There was an inverse correlation between virion (and cellular) hA3G levels by western blot and virion infectivity.

Neutralizing anti-IFN-γ antibody was used to decrease expression of A3G in Th1 cells (as seen in Figure 3). Incubation with neutralizing anti-IFN-γ antibody, concurrent with activation, reduced the expression of A3G in Th1 cells nearly 2 fold (relative to Th1 cells incubated with an isotype control antibody) (Figure 5C, open bars). Virions produced from Th1 cells with reduced hA3G had increased infectivity (Figure 5C, closed bars). Taken together, these data indicate that variation in infectivity of virions produced from cells is related to differences in hA3G expression.

## Discussion

In this study, we have shown that the expression and anti-HIV function of hA3G and hA3F vary with naïve CD4+ T helper cell differentiation to Th1 and Th2 effector cells. Cytokine polarization of naïve cells into Th1 and Th2 effectors had similar effects to transduction of naïve cells with Tbet or GATA3. In both cases, decreased expression of hA3G and hA3F was seen in Th2 cells relative to Th1 cells. These complementary methods demonstrate that the differences observed in the Tbet and GATA3 transduced cells were due to transcription regulated by those factors and not an artifact of over-expression. Such an opposing effect of differentiation on expression of hA3G and hA3F is consistent with earlier findings of opposing effects on the expression of several other genes in these two T helper subtypes [21],[36]. This hA3G and hA3F expression difference between Th1 and Th2 cells affected wild type, as well as *vif*-deleted, HIV-1 infectivity.

Expression of Tbet in naïve helper cells has been shown to lead to production of IFN-γ[Σζαβo, 2000 #35]. In turn, that IFN-γ can act in an autocrine manner on Th1 cells [34]. Extracellular neutralization of IFN-γ secreted by Tbet-transduced and control-transduced cells blocked basal and TCR-stimulated hA3G and hA3F expression. This is consistent with an autocrine effect of IFN-γ regulating hA3G and hA3F expression. GATA3 is known to inhibit the production of IFN-γ [37] and no effect was observed with neutralizing anti-IFN-γ antibody or TCR stimulation of GATA3-transduced cells. This may be due to a GATA3-mediated block to production of IFN-γ, or a direct effect of GATA3 binding to the hA3G and hA3F promoters. These possibilities remain to be directly tested.

We verified that the difference in expression in cytokine-derived T helper cells led to a biological difference: infectivity of HIV-1 virions produced from Th1 and Th2 effectors varied inversely with their relative levels of cellular and virion hA3G and hA3F. Removal of vif resulted in reduced infectivity of virions produced from both cell types. The greater reduction of infectivity of virions produced from the Th1 cells is consistent with the relative greater APOBEC3 levels in those cells. Over-expression of hA3G in Th2 cells reversed the relative decrease in virion hA3G and the consequent relative increase in infectivity of virions produced from Th2 cells. The magnitude of the effect of the ectopically-expressed hA3G is likely underestimated here, as not every cell in this population is expressing the transduced hA3G. In addition, reduction of hA3G in Th1 cells also correlated with an increase in infectivity. We used neutralizing anti-IFN-γ antibody to decrease hA3G expression in Th1 cells because shRNA against hA3G or nucleofection (for introduction of siRNA against hA3G) proved toxic to *in vitro*-derived Th1 cells, which are more prone to cell death than other cultured T cells [38]–[40].These results are consistent with the variation in virion infectivity being caused, at least in part, by the differences in cellular and therefore virion hA3G, rather than other effects of the cytokine derivation.

In this study, we observed reduction of infectivity associated with increased amounts of readily detectable virion hA3G without identification of any G-to-A hypermutation. Although hA3G and hA3F are cytidine deaminases, there is extensive evidence that hA3G also reduces HIV infectivity through other mechanisms that may be the major contributor to A3G's inhibition of reverse transcription [26],[33],[41],[42]. Previous studies that have observed hA3G-related hypermutation *in vitro* differed from the short term virus replication allowed here, and instead used prolonged serial passage of HIV in transformed cell lines over-expressing hA3G [43],[44]. Therefore, it is likely that the difference in infectivity based on cell source of virus observed here is due to the other antiviral activities of hA3G that are not measured by hypermutation.

A major issue concerning the role of hA3G and hA3F in HIV-1 pathogenesis is the question of whether *in vivo* variation in these cellular restriction factors affects replication of wild type (eg, *vif*-competent) HIV-1. Although high level over-expression of hA3G does impair replication of wild type HIV-1 in cell lines [4], more recent studies have not conclusively determined if there is a correlation between the variation in cellular hA3G expression observed across HIV-infected individuals' peripheral blood mononuclear cells and the plasma viral load in these subjects [29],[30],[32]. The present results clearly indicate that physiological variations in hA3G levels in primary cells are inversely correlated with hA3G content and infectivity of wild type virions. This more direct measure of biological relevance observed here supports the conclusions of earlier reports showing that greater A3G activity was associated with lower viral load set-point [32], and suggests that continued investigation of the effect of APOBEC3 restriction factors on *vif*-competent HIV-1 pathogenesis *in vivo* is warranted.

The present results are also consistent with earlier reports that HIV-1 spreads better through cultures of Th2 cells than Th1 cells [24]. This effect was most apparent in the prior studies using CXCR4 (X4) tropic viruses [25], such as the viruses used here, and not explained by differences in expression of that co-receptor between Th1 and Th2 cells. The present results suggest, however, that virions produced from Th2 cells may be relatively more infectious than those produced from Th1 cells because of their relatively lower hA3G content. In an earlier study [25], CCR5-tropic HIV replicated equally well in Th1 and Th2 cells. Th1 cells express higher levels of CCR5 coreceptor than Th2 cells [21],[22]. Indeed, X4-tropic viruses were chosen for study here to minimize possible difficulty in interpretation of opposing effects of both increased CCR5 co-receptor expression and increased A3G expression in Th1 cell cultures, though further investigation into how co-receptor tropism affects infectivity is certainly warranted. Moreover, the wide inter-individual variation in hA3G and hA3F expression in our results (a 14 fold range in hA3G protein expression in Th1 cells and a four fold range in Th2 cells) suggests that there may be polymorphisms in the regulatory regions of the APOBEC3 promoters [45], or in factors that can modulate hA3G and hA3F expression or function. We hypothesize that this variation in hA3G and hA3F may contribute to the wide variation of progression time to AIDS among different patients. The Th1/Th2 cell balance may also vary across individuals based on several factors. Autoimmunity may lead to a Th1 cell skewing and parasitic infections may cause aTh2 cell predominance. Our findings suggest that a shift in this balance prior to, or during, HIV-1 infection may lead to compounded pathogenic effects. Decreased relative expression of hA3G and hA3F in Th2 cells may lead to a greater rate of decrease in that cellular pool, decreasing CD4+ help to B cells for antibody production. Also, an individual's variation in Th1/Th2 balance may lead to differences in HIV-1 genetic variation due to hA3G- and hA3F-mediated sub-lethal cytidine deamination of viral genomes over repeated cycles of infection [46].

The present study indicates that the regulation of expression of hA3G and hA3F, and their functional effect on HIV-1 infectivity, depends on the cytokine-regulated differentiation state of CD4+ T helper cells. Further molecular characterization of signals that modulate hA3G and hA3F expression will be needed. The current results provide compelling evidence that increasing hA3G in primary T cells impairs HIV-1 replication despite the presence of Vif. This validates inducing higher hA3G expression as a novel strategy for prevention of infection and/or treatment of the *vif*-positive viruses present in infected humans.

## Materials and Methods

### Cells

Blood was obtained from healthy volunteers under a protocol approved by the Vanderbilt Institutional Review Board. PBMCs were isolated using Ficoll Hypaque (Amersham Biosciences). CD4+ cells were isolated by negative selection through magnetic separation using autoMacs (Miltenyi Biotec, Auburn, CA) or Robosep (StemCell Technologies, Vancouver, BC. Canada). Naïve cells were subsequently purified by staining with CD45RO-FITC and CD25-PE (BD Pharmingen, San Jose, CA) followed by sorting on a FACSAria (Becton Dickinson, San Jose, CA). For activation and expansion, naïve cells were plated in wells coated with an anti-CD3 antibody (OKT3; American Type Culture Collection, Manassas, Virginia, United States) in RPMI with 10% FBS supplemented with 1 µg/ml soluble anti-CD28 antibodies (BD Biosciences Pharmingen) and 50 U/mL human rIL-2 (obtained from Dr. Maurice Gately, Hoffmann - La Roche Inc. through the AIDS Research and Reference Reagent Program, Division of AIDS, NIAID, NIH) [47]. DMEM with 10% FBS was used to culture TZM-bl cells (obtained from Dr. John C. Kappes, Dr. Xiaoyun Wu and Tranzyme Inc. through the NIH AIDS Research and Reference Reagent Program, Division of AIDS, NIAID, NIH) [48].

### Transduction and T Helper Cell Differentiation

Naïve CD4+ T cells were differentiated by transduction with HIV derived lentiviral vectors expressing Tbet, GATA3, or a control vector at the time of activation [49],[50]. The vectors express GFP alone (control), or the transcription factor and GFP, from an IRES. After infection and activation, cells were expanded for 10 days. Following expansion, cells were sorted on a FACSAria for GFP expression.

To achieve Th1 cell differentiation using cytokine polarization, naïve CD4+ T cells were plated on anti-CD3 (OKT3) coated plates in RPMI supplemented with anti-CD28 antibodies, 0.5 µg/mL neutralizing anti-IL-4 antibody and 30 ng/mL recombinant IL-12. For Th2 cell differentiation by cytokines, naïve cells were cultured in media supplemented with 2.5 µg/mL neutralizing anti-IFN-γ antibody and 50 ng/mL recombinant IL-4. Cytokines and neutralizing antibodies were obtained from R&D Systems, Minneapolis, MN. The cells were expanded for 10 days and differentiation was confirmed by intracellular cytokine staining for IL-4-PE and IFN-γ-APC (BD Pharmingen, San Jose CA.) as previously described [49], as well as surface staining for CXCR3-PE and CRTh2-APC (BD Pharmingen, San Jose CA) [51]–[53]. To increase APOBEC3G expression in cytokine polarized Th2 cells, differentiating cultures were transduced with an APOBEC3G-expressing HIV derived lentiviral vector at the time of activation. The vector was constructed as other HIV derived lentiviral expression vectors previously described to express hA3G and HSA as a marker of transduction [50]. To reduce APOBEC3G expression in cytokine polarized Th1 cells, fully differentiated Th1 cells were activated for 48 hrs with CD3/CD28 coated beads (Invitrogen) in the presence of 5 µg and 10 µg anti-IFN-γ antibody (R&D Systems).

### Real Time PCR

Cytoplasmic RNA was isolated from cell pellets (Qiagen RNeasy, Valencia, CA). RNA was quantified by spectrophotometry on a GeneQuant Pro (Amersham Biosciences, Piscataway, NJ). RNA concentrations were normalized and TaqMan quantitative real-time RT-PCR was performed (Applied Biosystems Prism 7000 Sequence Detection System, Foster City, CA). Reverse transcription used hA3G and hA3F specific primers with the sequences 5′- GCGGCCTTCAAGGAAACC-3′ and 5′-TTTTAAAGTGGAAGTAGAATATGTGTGGAT-3′, respectively. The primer-probe set used for APOBEC3G real-time PCR was: forward: 5′-CTGCTGAACCAGCGCAGG-3′ reverse: 5′-GCGGCCTTCAAGGAAACC-3′ and probe: 5′-CTTTCTATGCAACCAGGCTCCACATAAAC-3′. The set for APOBEC3F was: forward: 5′-GCACCGCACGCTAAAGGA-3′, reverse 5′- TTTTAAAGTGGAAGTAGAATATGTGTGGAT -3′ and probe: 5′TTCTCAGAAACCCGATGGAGGCAATG-3′. Values are expressed as copies of target per million copies of GAPDH or calculated as fold change using the delta-Ct method [54].

### Western Blotting

Transduced or cytokine-derived T helper subtype cells were lysed in 50 mM HEPES, pH 7.4, 125 mM NaCl, 0.2% NP-40, 0.1 mM PMSF and EDTA-free protease inhibitor cocktail (CalBiochem, San Diego, CA). Protein concentrations were normalized based on results of a Bradford Assay (Bradford Assay reagent, Bio-Rad, Hercules, CA). Lysates were separated on a SDS-PAGE gel and proteins were subsequently transferred to a Trans-Blot nitrocellulose membrane (Bio-Rad, Hercules, CA). The membrane was then incubated with a polyclonal anti-APOBEC3G antibody [55], washed and probed with a goat anti-rabbit secondary antibody conjugated with Alexa Fluor 680 (Invitrogen Molecular Probes, Carlsbad, CA). Fluorescent signal was then measured using the Licor Odyssey system (LI-COR Biosciences, Lincoln, Nebraska). Membranes were subsequently probed with a monoclonal β-actin antibody (Sigma, St. Louis, MO) followed by a sheep anti-mouse secondary antibody conjugated with IR-Dye800 (Rockland Immunochemicals, Philadelphia, PA). APOBEC3G expression is expressed as fluorescent intensity (Relative Light Units, RLU) of APOBEC3G bands divided by the fluorescent intensity (RLU) of the β-actin band [56]. For quantification of virion packaged APOBEC3G, virions were concentrated by centrifugation of culture supernatants through a 20% sucrose cushion at 125,000×*g* for 45 minutes and normalized for their p24 content with viral lysis buffer [50 mM Tris (pH 8.0), 40 mM KCl, 50 mM NaCl, 5 mM Na_2_EDTA, 10 mM DTT and 0.1% (v/v) Triton X-100]. Lysates were blotted as described above with anit-APOBEC3G and an anti-HIV-1 capsid p24 antibody derived from the 183-H12-5C hybridomas (obtained from Dr. Bruce Chesebro and Dr. Hardy Chen through the NIH AIDS Research and Reference Reagent Program, Division of AIDS, NIAID, NIH [57]). Data are expressed as fluorescent intensity (RLU) of APOBEC3G bands divided by the fluorescent intensity (RLU) of the HIV-1 CA p24.

### Viruses and Infectivity

HIV-1 was produced by calcium phosphate transfection of 293T cells using NL4-3 (obtained from Dr. Malcom Martin through the NIH AIDS Research and Reference Reagent Program, Division of AIDS, NIAID, NIH) [58] and vif-deleted NL4-3 (a gift from the Chris Aiken Laboratory, constructed by Hevey and Donehower) [59]. After determination of the concentration of viral particles by HIV-1 CA p24 ELISA, 300 ng of p24-equivalents of HIV-1 were spinoculated (300×g, 30 min) on 1×10^6^ Th1, Th2, or TH2-A3G cells that had been activated by anti-CD3/CD28 coated beads (Invitrogen Dynal, Carlsbad, CA) for 60 hours [60]. Twelve hours after infection, cultures were washed twice with PBS. The cells were then resuspended in RPMI media containing 10 uM didanosine (Sigma, St. Louis, MO) and 25 uM zidovudine (Sigma, St. Louis, MO) to limit virus spread. After another 12 hrs in culture, supernatant fluids were collected for p24 antigen ELISA. Equal p24 concentrations of viral supernatant were then used to infect the TZM-bl indicator cells [48] and luciferase activity was determined in cell lysates 60 hours after infection (Bright-Glo Luciferase assay substrate, Promega, Madison, WI; TopCount scintillation counter, Packard/Perkin Elmer, Waltham, MA). Data are shown as RLU per nanogram p24 CA added.
